# The other side of variant transthyretin amyloidosis with polyneuropathy: psychosocial experience of members of Portuguese families with late onset of the disease

**DOI:** 10.1007/s12687-025-00776-5

**Published:** 2025-02-20

**Authors:** José D. Pereira, Andreia Santos, Eugenia Cisneros-Barroso, Intissar Anan, Marina S. Lemos, Milena Paneque

**Affiliations:** 1 CGPP - Centre for Predictive and Preventive Genetics, IBMC - Institute for Cell and Molecular Biology, University of Porto, i3S - Institute for Research and Innovation in Health, Porto, Portugal; 2 ICBAS - School of Medicine and Biomedical Sciences, University of Porto, Porto, Portugal; 3 Associação de Solidariedade Social “O Tecto”, Vila do Conde, Porto, Portugal; 4 Balearic Research Group in Genetic Cardiopathies, Sudden Death and TTR Amyloidosis, Health Research Institute of the Balearic Islands (IdISBa), Palma, Balearic Islands Spain; 5 Department of Public Health and Clinical Medicine, Umeå University, Umeå, Sweden; 6 Wallenberg Centre for Molecular Medicine, Umeå University, Umeå, Sweden; 7 FPCEUP - Faculty of Psychology and Educational Sciences, University of Porto, Porto, Portugal; 8 CPUP - Center for Psychology, University of Porto, Porto, Portugal

**Keywords:** Adult, Amyloidosis, Hereditary, Transthyretin-related, Genetic counseling, Portugal, Qualitative research

## Abstract

This study is the first to explore the psychosocial experience of members of Portuguese families with late-onset variant transthyretin amyloidosis with polyneuropathy (A-ATTRv-PN). Based on a constructivist worldview, this phenomenological investigation followed a qualitative approach by conducting eight interviews and analyzing qualitative data. The main results suggest that the psychosocial experience of the members of families interviewed is marked by: (a) a delayed awareness of the family disease (viz., in adulthood), (b) psychosocial impacts (viz., emotional and other impacts related to work, parenting, caregiving) experienced and anticipated in an adult phase of the life cycle, and (c) the use of approach strategies (e.g., seeking information about A-ATTRv-PN and seeking social support) and/or avoidance strategies (e.g., avoiding seeking information and talking to others about the condition) with a view to accommodating A-ATTRv-PN in personal and family life. These results differ from the life trajectories of members of Portuguese families with A-ATTRv-PN described previously and extend previous scientific evidence on the psychosocial experience of members of families where the disease typically appears late, contributing to further study on this topic and to the optimization of genetic counseling practices and health policies that respond to the psychosocial needs of members of Portuguese families with late onset of the condition. Future studies should continue to deepen our understanding of the psychosocial experience of this population to improve the clinical response provided to patients, families, and caregivers.

## Introduction

Variant transthyretin amyloidosis with polyneuropathy (A-ATTRv-PN), also known as familial amyloidotic polyneuropathy (FAP), is a rare multisystem disease with the predominant involvement of the peripheral nervous system (Adams et al. 2019). Although central nervous system involvement is also known (Sousa et al. 2021), the extent to that this is clinical or statistically meaningful is not clear. Prevalent worldwide, it is considered endemic in several regions, such as Portugal (Schmidt et al. 2018). A-ATTRv-PN is an autosomal dominant condition caused by the accumulation of amyloidogenic transthyretin in organs and tissues (Adams et al. 2019), which is mainly the result of the presence of the V30M (p.V50M) variant (Parman et al. 2016) in the transthyretin gene (Adams et al. 2019). Associated with this variant, and despite traditionally being thought of as an early-onset disease (< 50 years) in Portugal (Adams et al. 2021), Inês et al. (2018) reported a significant increase in the average age of onset and an increase in the representation of patients with late-onset A-ATTRv-PN (≥ 50 years) in the country, something more common in countries such as Sweden, France and Italy (Adams et al. 2021). In any case, to prevent the condition from progressing rapidly to death, patients diagnosed with A-ATTRv-PN may benefit from the early use of disease-modifying therapies. If, before 2018, liver transplantation for A-ATTRv-PN (Holmgren et al. 1993) was the primary treatment, this option has since been surpassed mainly by more effective drug therapies (Keller et al. 2025), including transthyretin kinetic stabilisers (such as tafamidis; Bulawa et al. 2012) and gene-silencing drugs (inotersen, patisiran, vutrisiran, and eplotersen; Qarni et al. 2024). However, regulating bodies for treatment of A-ATTRv-PN differ by country/regulating board (e.g., while in the European Union it is the European Medicines Agency, in the United States of America it is the Food and Drug Administration), so the availability of these drugs may be locally conditioned. On the other hand, genetic counseling in the context of presymptomatic testing (PST) is recommended for family members of people affected by A-ATTRv-PN (Obici et al. 2016).

Based, on the one hand, on the idea that knowledge of future disease risk through genetic testing should be managed within a context of family relationships, cultural beliefs, resources, as well as the wider healthcare and societal systems (Rolland and Williams 2005), and, on the other hand, on the concept of a systemic interaction between the condition and the family that evolves over time (Rolland 2012), A-ATTRv-PN can be conceptualized according to the different time at which it begins in the life cycle (childhood/adolescence, 0–20 years old; early-middle adulthood/childrearing years, 20–60 years old; and late adulthood, over 60 years old), which has significant biopsychosocial implications for these patients and their families (Rolland 2012; Rolland and Williams 2005).

The scientific research produced (e.g., Damy et al. 2022; Lopes et al. 2018; Magliano et al. 2021) has contributed to a better understanding of the psychosocial experience of A-ATTRv-PN and its implications for the lives of members of families with this disease. Specifically, members of Portuguese families with A-ATTRv-PN have been described as a population with substantial prior family contact with the condition, leading to the family being considered by these individuals as the main source of knowledge and learning about A-ATTRv-PN (Leite et al. 2016; Lopes et al. 2018; Paneque et al. 2019). According to Lopes et al. (2018), illness and death of a parent were frequent occurrences before young adulthood for individuals belonging to Portuguese families with A-ATTRv-PN. Other consequences in that period of life, and possibly related to it, included family disruption such as moving home, and being cared only by one parent or by another member of the family. Nevertheless, during childhood or youth, many individuals became caregivers, implying changes in family roles. Therefore, the disease and its implications have been represented as a significant psychosocial burden since childhood for members of Portuguese families with A-ATTRv-PN (Lopes et al. 2018). Instead, in countries where the condition typically appears later in the family (e.g., Sweden, France, and Italy; Adams et al. 2021), scientific evidence (e.g., Damy et al. 2022; Jonsèn 1998; Magliano et al. 2021) has mainly reported psychosocial impacts (viz., related to adult life structures such as work, parenting, caregiving) and their coping strategies in adulthood, a period of the life cycle with specific family developmental tasks (viz., adjusting the couple relationship with parenting, financial, and household demands, and realignment of relationships with extended family and grandparenting roles; Carter and McGoldrick 1999).

Thus, although the life trajectories of Portuguese families with A-ATTRv-PN have been described previously (Lopes et al. 2018), psychosocial research based on the time of clinical onset of the disease in the life cycle is scarce. Thus, taking into account the increase in the average age of onset and the representativeness of patients with a late onset of the condition in Portugal (Inês et al. 2018) and that different times of onset of the disease in the life cycle can have an influence on the biopsychosocial consequences felt by individuals and their families (Rolland 2012; Rolland and Williams 2005), a study on the psychosocial experience associated with the late onset of A-ATTRv-PN in members of Portuguese families can contribute to filling gaps and optimizing the health services that support these people in the context of genetic counseling. In fact, this study aims to explore the psychosocial experience of members of Portuguese families with late-onset A-ATTRv-PN, a topic that has not yet been specifically investigated in this population, to the best of our knowledge.

## Method

### Study design

Based on a constructivist worldview, this phenomenological research followed a qualitative approach by conducting interviews and qualitative data analysis processes suggested by Creswell and Creswell (2018) and Tesch (1990). This study design was selected to explore and understand the multiple meanings of the psychosocial experience of members of families with late-onset A-ATTRv-PN (i.e. where the age of onset of the disease in the index patient of the family is 50 years old or older), providing results with methodological integrity.

### Participants

Participants were recruited from health institutes (e.g., CGPP - Centre for Predictive and Preventive Genetics) and A-ATTRv-PN patient associations located in Portugal. An information sheet about the study and its objectives was circulated at these locations, inviting people to a face-to-face or telephone interview according to their convenience and availability. Those who showed an interest in participating were approached by telephone by JDP to clarify any doubts about the research and to schedule the interviews. The convenience sampling method was used to select the participants who were members of families with late-onset A-ATTRv-PN aged 18 or over and expressed their consent to participate.

The mean age of the participants was 55.38 years (*SD* = 17.51). All participants were of European ethnic origin and living in the northwest or central regions of Portugal. Additional sociodemographic information and participant data related to health and illness are described in Tables 1 and 2, respectively.

**Table 1:** Participants’ sociodemographic information

Sociodemographic characteristics	*n*	%	Participants
**Sex**			
Female	6	75	1, 2, 5, 6, 7, and 8
Male	2	25	3 and 4
**Age (in years)**			
26	1	12.5	6
39	1	12.5	5
43	1	12.5	2
58	1	12.5	3
65	1	12.5	1
67	1	12.5	4
68	1	12.5	7
77	1	12.5	8
**Education**			
Basic education	2	25	1 and 4
Secondary education	1	12.5	6
Higher education	5	62.5	2, 3, 5, 7, and 8
**Occupational status**			
Active	3	37.5	2, 5, and 7
Inactive	5	62.5	1, 3, 4, 6, and 8
**Marital status**			
Single	2	25	5 and 6
Married	2	25	2 and 4
Widowed	3	37.5	1, 7, and 8
Divorced	1	12.5	3
**Number of children**			
0	2	25	5 and 6
2	4	50	2, 3, 4, and 7
3	2	25	1 and 8

**Table 2:** Participant characteristics related to health and illness

Data related to health and illness	*n*	%	Participants
**History of psychological diseases**			
Yes	7	87.5	2, 3, 4, 5, 6, 7, and 8
No	1	12.5	1
**Diagnosis (and symptoms)**			
Carrier (asymptomatic)	2	25	1 and 7
Carrier (symptomatic)	3	37.5	3, 4, and 8
Not a carrier	2	25	2 and 5
No diagnosis	1	12.5	6
**Treatments**			
No treatment	5	62.5	1, 2, 5, 6, and 7
Undergoing treatments	3	37.5	3, 4, and 8
**Family history of A-ATTRv-PN**			
Age at onset of the condition in the family			
20–60 years old	6	75	1, 2, 3, 4, 5, and 6
> 60 years old	2	25	7 and 8
**Index patient status**			
Living with symptoms	3	25	2, 5, and 6
Died without symptoms	1	37.5	1
Died with symptoms	4	12.5	3, 4, 7, and 8
**Caregiver of someone with A-ATTRv-PN**			
Yes	4	50	2, 5, 6, and 8
No	4	50	1, 3, 4, and 7

### Procedure

Between January and February 2023, this study involved eight telephone interviews until saturation of the data relevant to the topic under study was reached, as postulated by Charmaz (2006). The semi-structured individual interviews included collecting sociodemographic and other information related to health (viz., history of psychological diseases and respective intervention) and the disease (viz., symptoms, diagnosis and treatments associated with A-ATTRv-PN, as well as multigenerational family history with the condition), followed by open and closed questions about the psychosocial experience associated with the late onset of A-ATTRv-PN in the family (viz., the psychosocial challenges related to the individual and family life cycle in symptomatic and nonsymptomatic phases of the disease). More specifically, they covered topics such as psychosocial adaptation to A-ATTRv-PN (e.g., family communication dynamics, psychosocial impacts, and coping strategies), belief systems (e.g., psychological representations of the condition), and psychosocial needs related to A-ATTRv-PN (e.g., social support). When other issues that emerged as salient were explored, the interviewer encouraged participants to clarify and elaborate on their arguments. The interviews lasted between 26 and 63 min, with an average time of 49 min.

#### Informed consent

was obtained from all the participants included in the study. All identifying information was removed from the transcripts by assigning identification codes to the participants. This action sought to ensure the confidentiality and anonymity of the data. The codes, which included the participant’s unique number (e.g., P1), are used in the Findings section to identify the source of the quotes. This research was approved by the Committee for Ethical and Responsible Conduct of Research of the Institute for Research and Innovation in Health.

### Analysis

All the interviews were audio-recorded with the consent of the participants and then transcribed verbatim into Portuguese by JDP. The transcripts were then analyzed by JDP following the qualitative data analysis process suggested by Creswell and Creswell (2018) as well as specific coding procedures proposed by Tesch (1990). After organizing and preparing the data for analysis, JDP read all the transcripts and began coding the information to generate themes, which are represented through a narrative passage to convey the analyzed results.

In order to ensure the production of findings with methodological integrity, multiple validity procedures (viz., triangulation of different participants’ perspectives, use of rich and dense descriptions to convey findings, presentation of discrepant information with the general perspective of the participants, spending prolonged time in the field under study, and participation of AS as peer debriefer) and reliability (viz., verification of transcripts and holding regular meetings between JDP and MP to discuss analysis) were incorporated into this research in accordance with the recommendations of Creswell and Creswell (2018), as well as Gibbs (2007), respectively.

## Findings

The analysis of the data showed that the psychosocial experience of the members of Portuguese families with late-onset A-ATTRv-PN interviewed is marked by: (a) a delayed awareness of the family disease, (b) psychosocial impacts experienced and anticipated at an adult stage in the life cycle, and (c) the use of strategies to accommodate the condition to personal and family life. These three themes are not mutually exclusive and represent fluid categories.

### Delayed awareness of the disease

All the participants in this study (e.g., P3 and P6) expressed that during their childhood/adolescence they did not notice patterns and attributes associated with A-ATTRv-PN in the family, which affected their own development of information related to genetic risk, the condition, and its biopsychosocial implications.

*P3: [During childhood/adolescence]*,* my father and mother took care of me. It was a normal process. I never noticed any manifestations of the disease*,* or any symptoms associated with A-ATTRv-PN. My father was a very active person and the development of the disease coincided with [his father’s] retirement. (…) I didn’t know what A-ATTRv-PN was. For me*,* the disease was in Póvoa [de Varzim]. I’m from Beira [region]. It [i.e. A-ATTRv-PN] wasn’t associated with Beira.*

*P6: [In childhood/adolescence]*,* it was my father and mother who looked after me. I never noticed any symptoms [in the family] related to A-ATTRv-PN. Everything was normal. (…) My father was the first person I contacted with A-ATTRv-PN. I didn’t know much about the disease. I had a vague idea that it wasn’t good*,* but I didn’t know what it was exactly. (…) I don’t know how it [i.e. the PST] works*,* nor do I know what the process is like. I have no idea.*

It was in adulthood that the participants were confronted with the development of the condition in the family and, in some cases, the loss of family members with A-ATTRv-PN, as P2 and P3’s speeches illustrate.

*P2: I was 38 years old [when the disease appeared in the family]. (…) My father complained of numbness and shocks in his legs and the doctor referred him to neurology [where he had a final diagnosis] two years later [in 2018]. The doctor said that the disease was progressing and that even medication didn’t help much. (…) This confirmed that my father wouldn’t have long [to live]. (…) Regardless of this*,* I realize that A-ATTRv-PN will be present for many years [in the family]*,* because I [also] have an uncle with the disease.*

*P3: I found out I had A-ATTRv-PN when I was 51 years old*,* at the same time as my father. (…) My father already had severe locomotion symptoms*,* ended up in a wheelchair*,* and died three months after [finding out he had the condition]. (…) I’m currently the only symptomatic person in the family*,* I’ve been taking tafamidis for seven years and what [the health professionals] tell me is that I’m the same as I was [when A-ATTRv-PN was diagnosed].*

### Psychosocial impact on adulthood

Participants declared that they had experienced a marked emotional transition when, as adults, they learnt about the existence of A-ATTRv-PN in the family and its biopsychosocial implications, as exemplified by the speeches of P5 and P6.

*P5: I was 32 years old [when A-ATTRv-PN appeared in the family]. It was a horrible time. I had a lot of anxiety. (…) [At that time]*,* I became more aware [of the condition] and it became more frightening*,* because I started to learn more [about A-ATTRv-PN]*,* which was and is a very bad disease. (…) [The process of carrying out the PST] was very bad. I couldn’t be productive at work or at home. I went out with people*,* but I was always thinking about it. I know I was very stressed*,* and I was completely daft at the time.*

*P6: I learnt [as an adult] from my father’s family doctor that I might develop A-ATTRv-PN. She told me very vaguely what the disease was. So*,* I tried to understand what it was [A-ATTRv-PN] and*,* of course*,* reading the symptoms*,* one person seems to have everything. At that early stage*,* I wondered a lot about what it would be like*,* what it would be like if I also had [the condition] and who was going to look after my father… At the time*,* I was shocked by what I read. (…) [At that stage]*,* I felt like I had a lot of nightmares. I had nightmares about my father falling and hurting himself. I had this constant fear.*

After the initial impact, participants (e.g., P6 and P7) reported experiencing and anticipating psychosocial impacts related to the structures of adult life (e.g., work, parenting, and caregiving).

*P6: My father is no longer independent. I stopped working to look after him. I had no other choice. He’s suffering so much that the least I can do is try to ease what he’s feeling. (…) Despite this*,* I still manage to have a few moments to myself. (…) Of course*,* I often can’t [go out with friends]*,* because he needs me for lunch and dinner. (…) Even when I do go out*,* I’m always afraid [that the father will fall when he gets up to do something] (…) Psychologically*,* there are complicated days when I think about tomorrow and what’s going to happen.*

*P7: I was 61 years old when I found out that I had A-ATTRv-PN. At the time*,* I had two pregnant daughters*,* and my husband was terminally ill [from another disease]. (…) The biggest shock was when I went to [Hospital] Santo António and saw very young people [with the condition]. I immediately thought of my daughters and the grandchildren I already had. (…) Knowing late on that I had A-ATTRv-PN made my life lighter. If I’d known when I got married or got pregnant*,* I wouldn’t have had the psychological structure to cope with the disease.*

### Disease accommodation strategies

Faced with the psychosocial impacts in adulthood, most of the participants (e.g., P3 and P4) declared that they had mainly used approach strategies (e.g., seeking information about A-ATTRv-PN and seeking social support) to accommodate the condition in their personal and family lives.

*P3: When I learnt that I might have A-ATTRv-PN*,* I started reading and finding out about it*,* always making sure to consult reliable sources. Then I started looking for Facebook pages like that of the Brazilian Association of A-ATTRv-PN. In the meantime*,* I also started contacting people in New Zealand*,* Ireland*,* and Venezuela to try and find out more [about the condition]. (…) I keep up to date with what’s going on. I try to stay informed so that I can keep abreast of developments in the disease.*

*P4: My friends know about my disease and are very supportive. When I sometimes go to the cemetery with them*,* they often come to support me because of the difficulties I have in getting around. I also go walking with them. They know that I have A-ATTRv-PN and that my step is not their step*,* but they accompany me anyway. (…) My wife is first-rate. She accompanies me everywhere (…) All this helps me to live with the disease better.*

Even so, other participants expressed having used avoidance strategies (e.g., avoiding seeking information and talking to others about A-ATTRv-PN) to deal with the experienced and anticipated impacts related to the condition, as P1’s speech illustrates.

*P1: I’ve never thought*,* I don’t even think about what I think and feel about A-ATTRv-PN. I think that the more you think about it*,* you start to have symptoms without [actually] having them. I try to forget that the disease exists. I don’t even try to talk about it [with others]*,* so that I don’t think too much [about the condition] and then get worse. [And in the future]*,* that’s my idea*,* not to think about it and not to talk about it.*

## Discussion

This study, the first to report specificities of the psychosocial experience stated by members of Portuguese families with late-onset A-ATTRv-PN, differs from the life trajectories of members of Portuguese families with A-ATTRv-PN described previously (Lopes et al. 2018) and extends previous scientific evidence on the experience of members of families where the disease typically appears later (e.g., Damy et al. 2022; Jonsèn 1998; Magliano et al. 2021). The main results suggest that the psychosocial experience of the members of families interviewed is marked by: (a) a delayed awareness of the family disease, (b) psychosocial impacts experienced and anticipated in adulthood, and (c) the use of strategies to accommodate the condition to personal and family life.

Firstly, and instead of the life trajectories previously described in the Portuguese population with the disease (Lopes et al. 2018), the participants in this study expressed that their childhood/adolescence was characterized by the absence of patterns and attributes associated with A-ATTRv-PN in the family (e.g., illness or death of a parent). In fact, the knowledge about risk, the condition and its biopsychosocial implications arising from previous family contact with A-ATTRv-PN, reported by several studies in Portugal (e.g., Leite et al. 2016; Lopes et al. 2018; Paneque et al. 2019), differs from the discourses of the members of Portuguese families with late onset of the disease interviewed. For these participants, it was in adulthood that, at the same time as experiencing the main family developmental tasks (e.g., adjusting the couple relationship with parenting, financial, and household demands, and realignment of relationships with extended family and grandparenting roles; Carter and McGoldrick 1999), they came into contact with their own and/or their family’s development of A-ATTRv-PN and its consequences (e.g., loss of a parent). As advocated by Rolland (2012) and Rolland and Williams (2005), this postponement of the psychosocial experience of the disease to later ages, associated with the increase in the average age of onset and the representativeness of patients with a late onset of A-ATTRv-PN in Portugal (Inês et al. 2018), has significant psychosocial impacts for these patients and their families.

Allied to a later contact with A-ATTRv-PN, members of families interviewed stated that they experienced a marked emotional transition when, as adults, they learnt about the existence of the condition in the family and its biopsychosocial implications. While an undercurrent of worries and fears about vulnerability to A-ATTRv-PN can emerge from childhood in members of families where the disease developed earlier (Lopes et al. 2018), the psychosocial experience of the members of Portuguese families with late-onset A-ATTRv-PN interviewed was mainly marked by a period of scarce involvement with the condition during childhood/adolescence that translated into a sudden emotional impact as they came into contact with A-ATTRv-PN and faced it in adulthood, which is in line with the postulate by Rolland and Williams (2005) and results reported in members of French (Damy et al. 2022) and Italian (Magliano et al. 2021) families with the disease. Nevertheless, the late onset of A-ATTRv-PN in the family caused psychosocial impacts (e.g., related to adult life structures) in participants in this study. Specifically, following the postulates of Rolland (2012) and Rolland and Williams (2005), while members of families who came into contact with the condition earlier in life may have experienced psychosocial impacts earlier in life and therefore be more likely to prepare for the future (Lopes et al. 2018), members of families with late-onset A-ATTRv-PN may be challenged to adapt their ongoing or already established life projects (e.g., related to work, parenting, and caregiving), as they have designed their lives in a less restrictive way previously. These data are in line with the results reported in previous studies with members of French (Damy et al. 2022) and Italian (Magliano et al. 2021) families, populations where the disease typically appears later. Even so, as Rolland (2012) and Rolland and Williams (2005) have already postulated, it is the quality of the fit between the psychosocial challenges caused by A-ATTRv-PN, on the one hand, and the functioning and resources of the support contexts, on the other, that may determine successful versus dysfunctional coping and adaptation to the condition.

Faced with the psychosocial impacts in adulthood and the scarce family psychosocial experience with the disease, participants in this study mainly used approach strategies associated with seeking information about A-ATTRv-PN from sources outside the family (viz., using data published on the Internet and Facebook communities of members of families with the condition), differing from the results reported by Leite et al. (2016) and Paneque et al. (2019), who considered the family to be the main source of knowledge and learning about A-ATTRv-PN for members of Portuguese families with the disease. On the other hand, the search for social support in the family environment and/or in friendships to optimize well-being, reported by participants in this study, is in line with results reported in previous studies with members of French (Damy et al. 2022) and Italian (Magliano et al. 2021) families. However, accommodating the condition to a life structure with ongoing or already established personal and family projects may become too challenging for certain members of Portuguese families with late-onset A-ATTRv-PN, so the use of avoidance strategies (e.g., avoiding seeking information and talking to family and friends about the disease), already reported in an investigation by Jonsèn (1998) with Swedish patients, may be the only viable way for these people to deal with the psychosocial impacts experienced associated with A-ATTRv-PN. On the other hand, the use of certain coping strategies may be influenced by key beliefs related to health and illness (e.g., mind–body relationship, control, and mastery associated with A-ATTRv-PN) built up in the development of the disease-individual-family triad (Rolland 2012).

### Strengths and limitations

The results reported in this study, which contribute to filling gaps in scientific evidence on the psychosocial experience with late-onset A-ATTRv-PN in a population with specific developmental tasks (Carter and McGoldrick 1999), should be carefully read and interpreted due to the presence of limitations inherent to research practice. Although, following the recommendations of Creswell and Creswell (2018) and Gibbs (2007), various validity and reliability procedures have been implemented in order to ensure the methodological integrity of the data, subsequent studies may incorporate additional analytical processes (e.g., conducting follow-up interviews with study participants and providing them with an opportunity to comment on the results, as well as introducing intercoder agreement processes), further strengthening the validity and reliability of their findings. Since the purpose of this research is restricted to the specific description of the psychosocial experience of members of Portuguese families with late-onset A-ATTRv-PN in a particular spatiotemporal context, readers should bear this in mind when considering the transferability of these results to other populations, diseases, and contexts. Nevertheless, it should be borne in mind that the sample studied included only members of families with late-onset A-ATTRv-PN who, on their own initiative, were willing to participate in the study, so further research could verify the generalizability of the data reported to the population of members of Portuguese families with A-ATTRv-PN in general and in other countries.

### Implications

The results presented highlight the importance of future studies continuing to delve deeper into the psychosocial experience of members of families with late-onset A-ATTRv-PN, since this is a population with an increasing representativeness of patients in Portugal and in which psychosocial research is scarce. Specifically, studies on the psychosocial experience of different patterns of the condition and on the role of key variables in the family system can contribute to filling gaps in the scientific evidence this population. It is also suggested that community policies to raise awareness of A-ATTRv-PN (and its different forms of onset) among the Portuguese population be strengthened, particularly among local health services, A-ATTRv-PN patient associations, and schools. The inclusion of genetic counseling professionals in multidisciplinary teams within these services, particularly in non-endemic areas of the disease in the country (e.g., Beira region), could also help to improve the informational and psychosocial issues associated with A-ATTRv-PN, especially in the way that these professionals are able to gather multigenerational family data and provide the most up-to-date scientific evidence on the condition, as well as providing information about useful coping and adaptation skills tailored to (a) the psychosocial demands on these affected families over time, (b) living with specific genetic-risk information, and (c) the fit between these psychosocial demands and the strengths and vulnerabilities of each family. Nevertheless, this multidisciplinary approach can cover the medical needs of this population in a timely manner (avoiding delays in diagnosis and the necessary treatment for a disease with therapies capable of slowing down its development), as well as improving the referral of these people to referral centers and hospital units. Finally, taking psychosocial factors into account and developing the skills of genetic counselors, psychologists, and other health professionals to meet the needs of patients, families and caregivers dealing with the different forms of onset of A-ATTRv-PN in nonsymptomatic and symptomatic phases is an essential factor for health services in this genomic era.

## Conclusion

This study reports the first in-depth description of the psychosocial experience of members of Portuguese families with late-onset A-ATTRv-PN. Given the complex challenges posed to families with the condition and to health systems, this research can provide important data for optimizing genetic counseling practices and health policies that respond to the specific needs of this population. Nevertheless, and given that different moments of onset of A-ATTRv-PN in the life cycle can have an influence on the biopsychosocial consequences felt by individuals and families with the disease, it is of the utmost importance to continue to deepen our understanding of their psychosocial experience with a view to improving the clinical response provided to patients, families, and caregivers.

## Data Availability

The participants in this study did not give consent for their data to be shared publicly, so supporting data is not available.

## Declarations

### Data transparency

José D. Pereira, Andreia Santos, Eugenia Cisneros-Barroso, Intissar Anan, Marina S. Lemos and Milena Paneque declare that all data support their published claims and comply with field standards.
